# Impact of partially and fully closed eaves on house entry rates by mosquitoes

**DOI:** 10.1186/s13071-018-2977-3

**Published:** 2018-07-03

**Authors:** Monicah M. Mburu, Malou Juurlink, Jeroen Spitzen, Paula Moraga, Alexandra Hiscox, Themba Mzilahowa, Willem Takken, Robert S. McCann

**Affiliations:** 1 Wageningen University and Research, Wageningen, The Netherlands; 2 College of Medicine, University of Malawi, Zomba, Malawi; 3 Centre for Health Informatics, Computing and Statistics (CHICAS), Lancaster Medical School, Lancaster University, Lancaster, UK; 4 MAC Communicable Diseases Action Centre, College of Medicine, Blantyre, Malawi

**Keywords:** House improvement, Eaves, Malaria vectors, House entry, *Anopheles*, Culicines, Vector control

## Abstract

**Background:**

Most people infected with malaria acquire the infection indoors from mosquito vectors that entered the house through open eaves, windows and doors. Structural house improvement (e.g. closed eaves and screened windows) is an established method of reducing mosquito entry. It could be complementary to other interventions such as insecticide-treated bed nets (ITNs) for malaria control because it covers and protects all individuals in a house equally. However, when implemented at a large scale, house improvement may not be employed optimally. It is therefore critical to assess whether partial house improvement will have any effect on mosquito house entry. We investigated the effect of partial and complete eave closure on the house-entry rates of malaria vectors and other mosquitoes in southern Malawi.

**Methods:**

The study was conducted for 25 nights in May-June 2016. Twenty-five traditional houses were modified according to five treatments: fully closed eaves, three different levels of partially closed eaves, and open eaves. All houses had fully screened windows and closed doors. Host-seeking mosquitoes were sampled inside these houses using Centers for Disease Control and Prevention (CDC) light traps. The effect of open eaves *versus* partial or complete eave closure on the number of mosquitoes trapped inside the house was estimated using a generalized linear mixed model fitted with Poisson distribution and a log-link function.

**Results:**

House entry by malaria vectors was 14-times higher in houses with fully open eaves compared to houses with fully closed eaves adjusting for wall-type, number of people that slept in the house the previous night, cooking locations and presence of livestock. Houses with four small openings had 9 times more malaria vectors compared to houses with fully closed eaves. The catches of culicine mosquitoes caught in houses with fully closed eaves were not different from those caught in houses with the other treatments.

**Conclusions:**

Closed eaves resulted in fewer malaria vectors in houses, with differences depending on the degree of eave closure. The ability of malaria vectors to locate any remaining entry points on improved houses, as demonstrated here, suggests that quality control must be an important component of implementing house improvement as an intervention.The lack of effect on culicine mosquitoes in this study could reduce acceptance of house improvement, as implemented here, by household residents due to continued nuisance biting. This limitation could be addressed through community engagement (e.g. encouraging people to close their doors early in the evenings) or improved designs.

## Background

Malaria continues to place a heavy burden on communities living in malaria endemic areas, in spite of promising declines in malaria globally due to the use of insecticide-treated bed nets (ITNs), indoor residual spraying (IRS) and effective drug therapy [1]. In endemic regions of Africa, where 90% of cases and deaths from malaria occur [2], indoor biting by malaria vectors still plays a prominent role in malaria transmission [3–5] and the structural design of houses affects the entry of malaria vectors into residences. Houses with modern features (e.g. closed eaves, screened doors and windows, and ceilings) can provide the first line of defense against bites from infected malaria vector mosquitoes, whereas houses without these features have been associated with increased numbers of mosquitoes indoors [6–8] and higher levels of malaria [9–12]. Open eaves are significant entry points into houses for malaria vector species in Africa [13–15] and are therefore recognized as a risk factor for malaria.

Most studies looking at house design and mosquito entry (or malaria) have been observational studies of incremental improvements in house design that occur coincidentally with socioeconomic improvements over time [10]. In addition to those studies, others have tested the effect of deliberate structural modifications, also known as house improvement, as a direct intervention to block mosquito entry using materials such as netting, papyrus reeds, sand, rubble and concrete. These studies have associated house improvement with fewer mosquitoes entering homes [16–18] and reduced anaemia prevalence in children [16].

Modern house features have been viewed favourably by residents because of their perception that these features reduce mosquito bites [17, 19–21], with the primary concerns being the costs of these features and the potential for increased indoor temperatures [17, 19]. Additional benefits of house improvement as an intervention against malaria include: equal protection is offered to all individuals in a house, no daily action from the end user is required, it is technologically simple and it does not require insecticides in principle.

These advantages, together with the spread of insecticide resistance threatening the efficacy of ITNs and IRS [22, 23], have led to a renewed interest in the broad concept of house improvement as an intervention and a need to address key questions about specific aspects of the intervention related to the effectiveness of particular features, safety, acceptability and implementation [24, 25]. As with any health intervention, measuring the percentage of the population effectively covered by house improvement will be important for understanding the effectiveness of the intervention in both trial settings [26] and on a larger scale (e.g. as programmes implement house improvement at a district or national scale). Here, we refer to the malERA Consultative Group on Health Systems and Operational Research and their definition of effective coverage, which goes beyond simple access to an intervention to also include provider compliance and client adherence [27, 28]. In the context of house improvement, compliance could be measured in terms of the number and size of any remaining gaps in the housing structure following implementation. While the goal of implementation would be to leave zero gaps for mosquito entry, in real-world settings this would not be the case for 100% of houses with access to house improvement. Therefore, it will be important to understand the extent to which houses with remaining gaps for mosquito entry following implementation of these modifications would still provide any effective protection from mosquito bites compared to fully improved houses. The aim of this study was to assess differences in partial or complete closure of the eaves on house-entry rates by anopheline and culicine mosquitoes in a randomized field experiment.

## Methods

### Study site

The study was conducted in Chikhwawa District, southern Malawi, which lies along the lower Shire valley. This area experiences a single rainy season from November through to April. The main malaria vectors prevalent in the region are *Anopheles gambiae* (*s.s.*), *An. funestus* and *An. arabiensis* [29, 30]. Malaria transmission occurs throughout the year with rates intensifying during the rainy season. Malaria parasitemia in children under five years of age in this region varies seasonally between 11–40% [31].

**Fig. 1:**
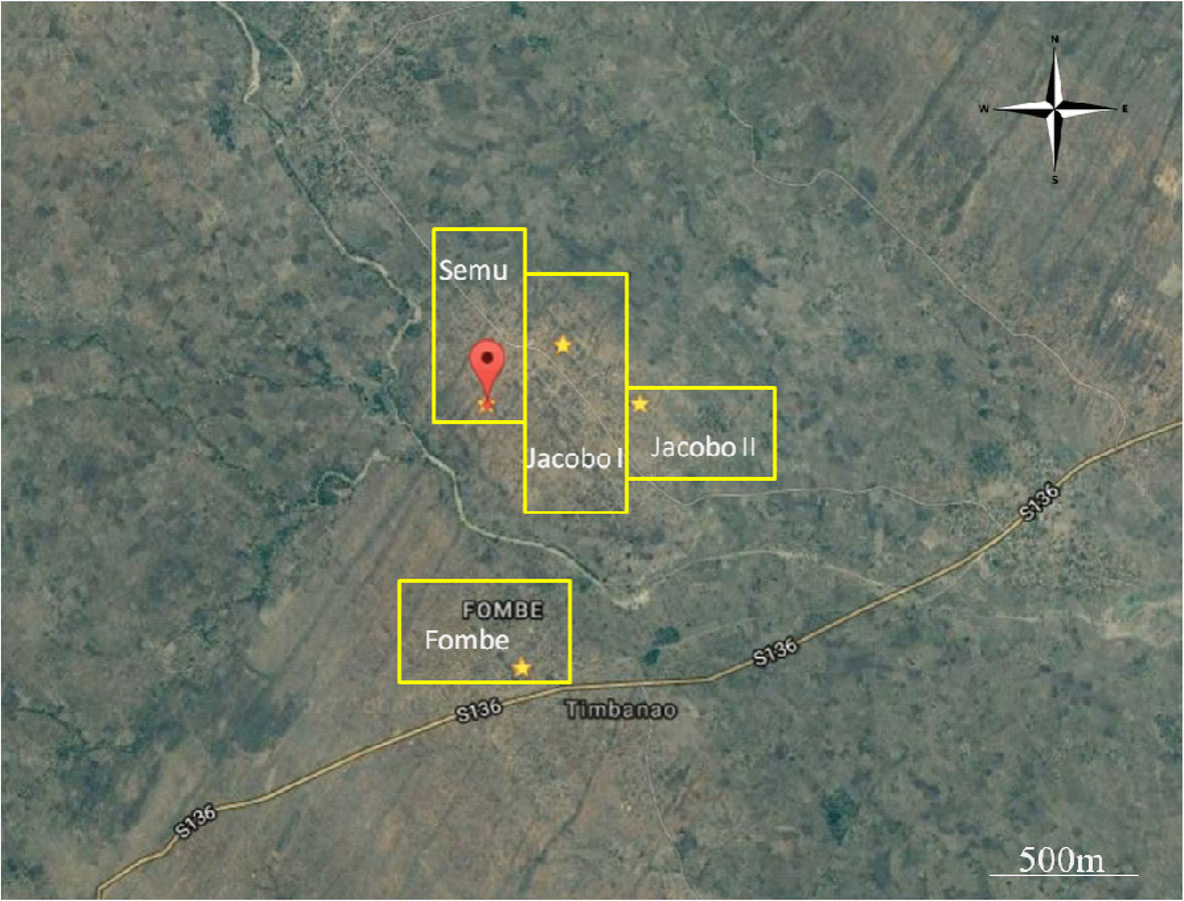
Geo-location of the four villages selected for the study. Fombe: 16.06962496, 34.73430784; Jacobo I: 16.0532887, 34.7365262; Jacobo II: 16.05628311, 34.74051196; Semu: 16.05625729, 34.73250272

**Fig. 2:**
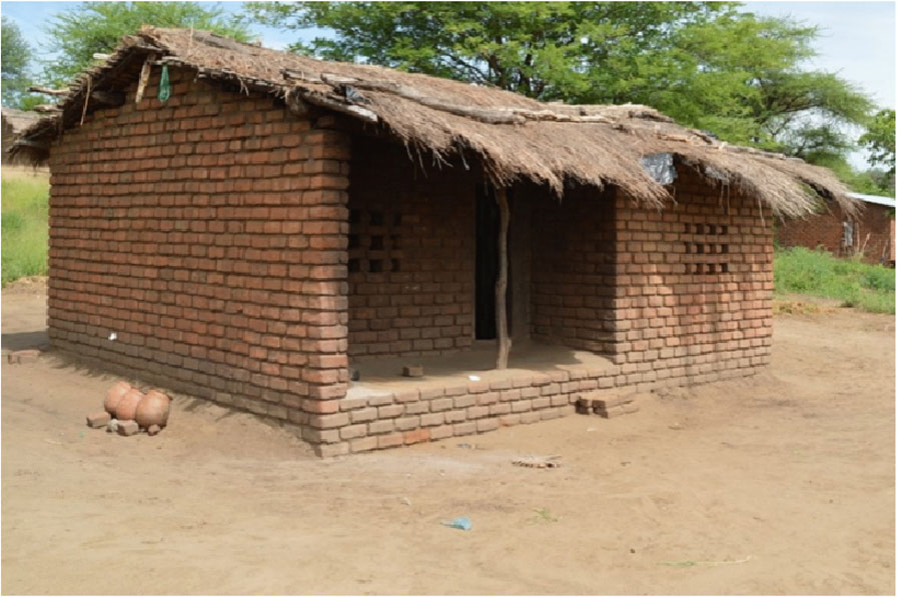
Photograph of a typical house in the study area

Four neighbouring villages in Chikhwawa District (Fombe, Jacobo I, Jacobo II and Semu) were identified for the study (Fig. 1), allowing for random selection of houses separated by a distance of 25m away from each other. The combined population of the villages was 4740 (personal communication, secretary-group village head). The area is relatively flat (i.e. little topographic relief), with two seasonal streams. Farming subsistence crops and small-scale cash crops is the primary means of occupation in the study area. Houses in the selected villages are typical for the region. The general house design consists of four walls in a rectangular arrangement with a two-sided roof oriented along the long axis of the house (Fig. 2). House walls are typically constructed with either sun-dried or fire-baked bricks, and roofs are made with either grass thatch or corrugated sheet metal. Most houses have one door, two to four square windows, and either open or closed eaves.

### House selection

The study included 25 houses. The local leaders (i.e. village chiefs) provided a list of 100 houses across the four villages separated by a distance of ≥ 25 m that fit the following criteria: open eaves, open windows, gaps around the doors, and grass thatched roofs. From these, twenty-five houses were randomly selected for enrolment into the study (6 from Fombe, 7 from Jacobo I, 7 from Jacobo II, and 5 from Semu).Prior to enrolment, we applied further inclusion criteria such that every house would be at least 20 m away from cattle sheds and within a range of 100 m from any mosquito breeding habitat. The houses that did not meet the inclusion criteria were replaced with the nearest neighbouring house that met all inclusion criteria. The geo-location of each house was recorded at enrolment.

### Treatments

**Fig. 3:**
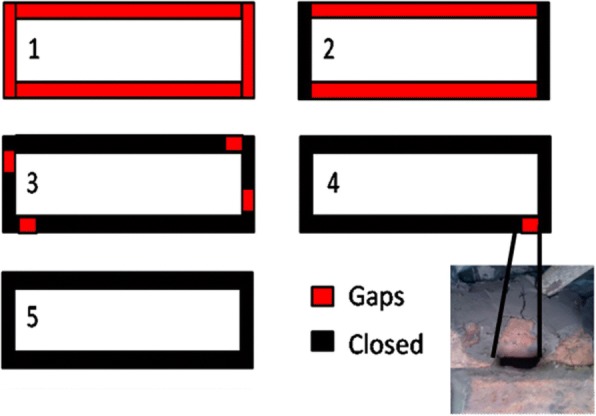
Design of the five treatments assigned to the sets of five houses

The five treatments in this study were: fully closed eaves, eaves with a single 5× 1cm opening (hereafter referred as a single small opening), eaves with four 5× 1cm openings (hereafter referred as four small openings), eaves with two long sides open and houses in which the eaves were open on all four sides (Fig. 3). Treatments were assigned randomly to each house using a random number generator in Microsoft Excel, with five houses being assigned to each treatment. For all 25 houses, all the gaps in walls were closed with muddy soil, gaps in the doors were closed with wooden planks and windows were closed with wire gauze. Small apertures between the window frame and the wall were filled in with mud. For houses with partial and complete eave closure, a combination of bricks and muddy soil were used for eave closure. Local builders and carpenters were hired to perform the house modifications which were checked for quality by the researchers at completion. The householders provided muddy soil, while the researchers provided the wire gauze for screening the windows and some bricks to close the larger openings. From our observations, the grass thatched roofs were intact, with the exception of one house where the roof had some openings. The owner of this house repaired the roof by filling in the openings with more grass.

### Mosquito sampling

Mosquito sampling was carried out for four nights a week for a total of 25 nights from 12 May to 24June 2016. Centers for Disease Control and Prevention (CDC) light traps were used to sample the mosquitoes inside all houses. The traps were powered by 6V batteries and operated from 17:00 h (15 min before sunset at that time of year) until 7:00 h (1 hour after sunrise). In each house, the trap was hung with the fan at 150cm above the ground, at the foot end of a bed in which a person was sleeping under a bed net [32, 33]. The bed nets were owned by the household. Every morning after a night of sampling, chloroform was used to immobilize the mosquitoes caught in the traps. The mosquitoes were then transferred into an Eppendorf tube containing a silica gel desiccant, and transported to the laboratory for morphological identification.

During mosquito collections, brief interviews were conducted with householders to obtain data on house parameters such as the number of people that occupied the house the previous night, livestock that stayed within 20 m of the house the previous night, wall type, floor type, door type and cooking locations. The following represent the categorizations: door type as wood and reed; floor type as dirt/mud/dung/sand; wall type as sun dried bricks and fire baked bricks; cooking location as inside the house, on the veranda, outside but within 2m of the house and outside more than 2m away from the house. Data were recorded on a tablet computer using Open Data Kit [34].

### Mosquito identification

All mosquitoes were identified morphologically as either anophelines or culicines. Anophelines were further classified as either *Anopheles gambiae* (*s.l.*), *An. funestus* or *An. coustani* using the dichotomous key published by Gillies & Coetzee [35]. There was no further classification of the culicines beyond the subfamily level. Females from the *An. gambiae* (*s.l.*) species complex were further identified to species level using polymerase chain reaction (PCR) [36].

### Data analysis

The effect of eave closure on the number of mosquitoes caught indoors was tested using a generalized linear mixed model fitted with a Poisson distribution and a log-link function. House identification number was included as a random effect in the model to account for the repeated measures by house. The kind of livestock that stayed within 20m of the house the previous night, the cooking location, wall type and the number of people who slept in the house the previous night were included as covariates in the model. Livestock comprised of cattle, goats, sheep, chicken and pigs. Sheep and pigs were excluded from the analysis because of the low number of houses with either animal (≤ 6). Similarly, floor and door types were excluded from the analysis because all the floors were made of mud; doors were made of wood in 24 houses while in the remaining house the door was made of reed. All analyses were performed using R, version 3.3. The primary outcome was the number of female malaria mosquitoes (hereafter referred as anophelines) caught with a CDC light trap per house, per night. Due to the low number of anophelines caught, count data for all anopheline species were pooled per treatment and day for statistical analysis. Secondary outcomes were the number of culicine females and the number of culicine males caught per house, per night. Fully closed eaves served as the reference in our analysis. Pairwise comparisons were performed with the Dunnett’s test to compare each of the treatments to the reference treatment, fully closed eaves.

## Results

Combined across all treatments, a total of 777 mosquitoes were collected over 625 trap-nights. Of these, 48 were female anophelines, 6 were male anophelines, 466 were female culicines, 248 were male culicines and 9 were unidentifiable. Of the female anophelines, 47 were *An. gambiae* (*s.l.*) and one *An. coustani*. Thirty-six and two of the female *An. gambiae* (*s.l.*) mosquitoes were identified to species level as *An. arabiensis* and *An. gambiae* (*s.s.*), respectively. The remainder (*n* = 9) could not be identified further because they failed to amplify. Abdominal status of the female anophelines included: unfed, 85.42% (*n* = 41); and fed, 14.58% (*n* = 7). No gravid or semi-gravid malaria vectors were caught. Abdominal status of female culicine mosquitoes trapped included: unfed, 97.21% (*n* = 453); fed, 2.58% (*n* = 12); and semi gravid, 0.21% (*n* = 1).

**Fig. 4:**
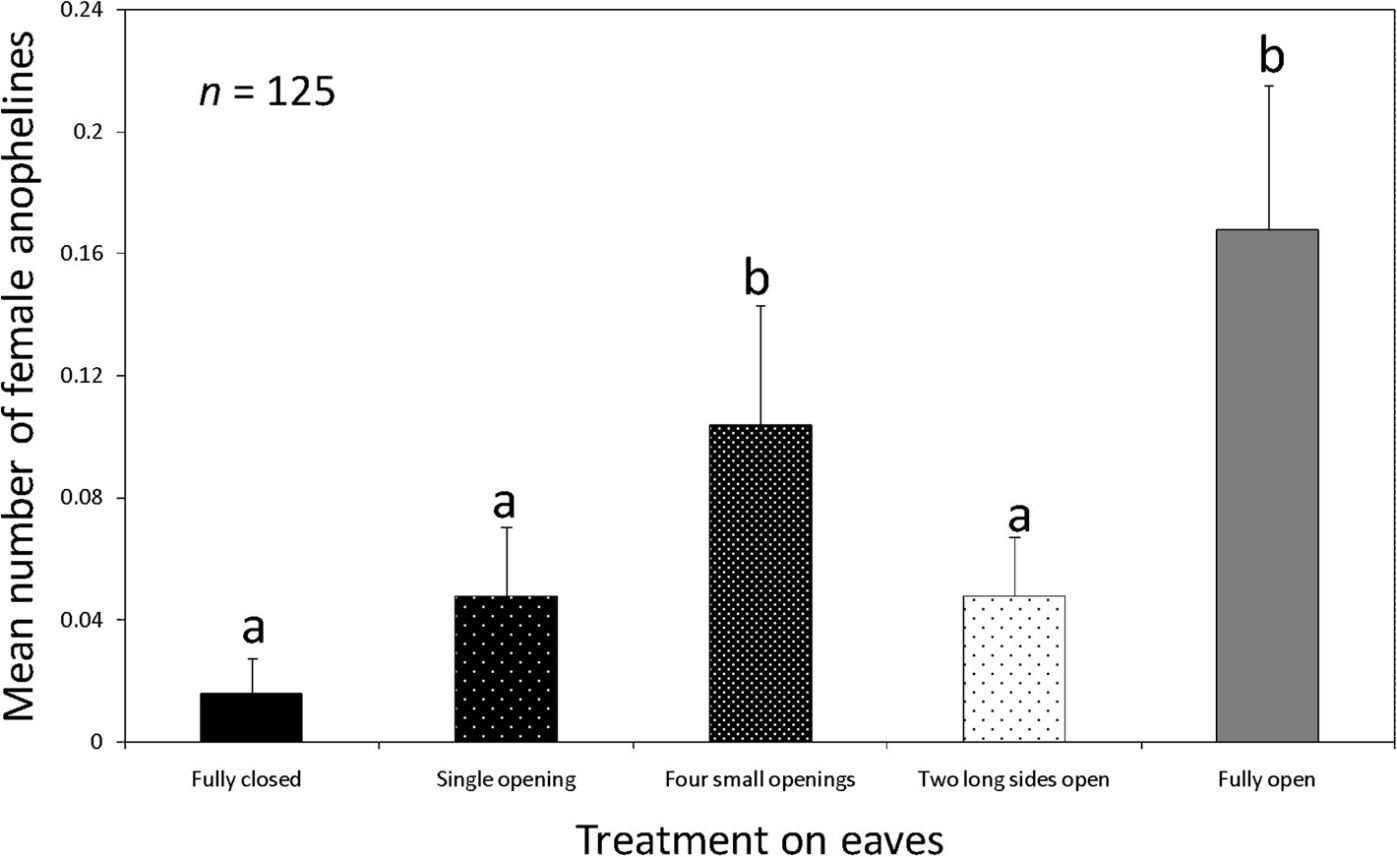
Mean number of female anophelines caught indoors with CDC light traps in houses where eaves were fully closed, had a single small opening, four small openings and fully open. Bars with different letters denote significant differences in the number of mosquitoes trapped. *n* = 125 trap nights for each treatment

The catches of female anophelines per treatment were: fully closed eaves, 4.16% (*n* = 2); eave with a single small opening, 12.5% (*n* = 6); eave with four small openings, 27.08% (*n* = 13); eave with two long sides open, 12.5% (*n* = 6); and open eaves: 43.75% (*n* = 21). Catches in houses with fully closed eaves were significantly lower than catches in houses with four small openings (Risk ratio, RR = 8.83, 95% CI: 1.16–67.14, *Z* = 2.105, *P* = 0.035), and with completely open eaves (RR = 14.16, 95% CI: 2.05–97.91, *Z* = 2.687, *P* = 0.007). Catch sizes of female anophelines caught in houses with fully closed eaves were similar to those in houses with a single small opening in the eave (RR = 4.38, 95% CI: 0.59–32.46, *Z* = 1.444, *P* = 0.149) and two long sides open (RR = 5.41, 95% CI: 0.72–40.40, *Z* = 1.645, *P* = 0.10) (Fig. 4). Pairwise comparisons between houses with fully open eaves and fully closed eaves showed that the female anopheline catches were different (*Z* = 2.687, Adjusted *P* = 0.022).

**Fig. 5:**
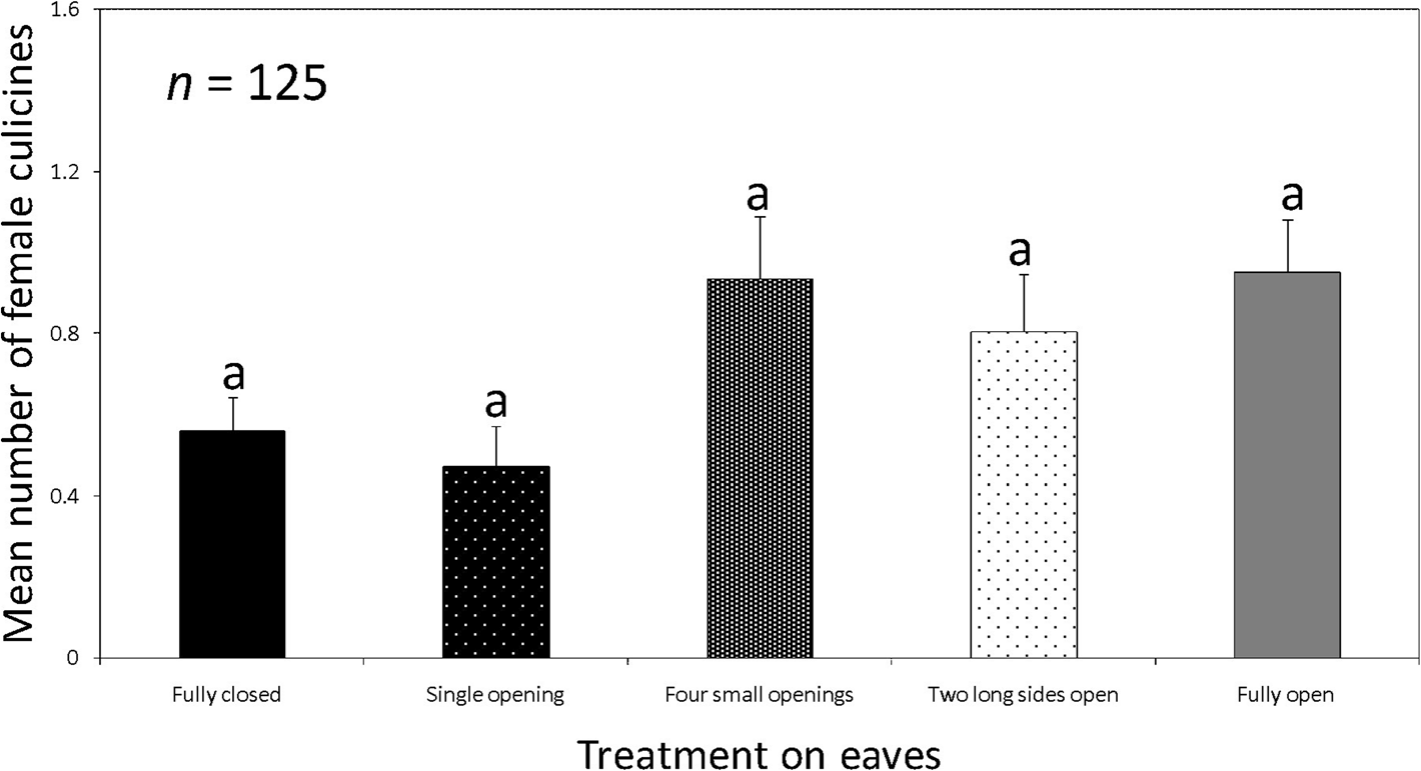
Mean number of female culicines caught indoors with CDC light traps in houses where eaves: were fully closed, had a single small opening, four small openings and fully open. Bars with same letters denote similarities in the number of mosquitoes trapped. *n* = 125 trap nights for each treatment

The catches of female culicine mosquitoes per treatment were: fully closed eaves, 15.02% (*n* = 70); eave with a single small opening, 12.66% (*n* = 59); eave with four small openings, 25.11% (*n* = 117); eave with two long sides open, 21.67% (*n* = 101); and open eaves, 25.54% (*n* = 119). Catch sizes of female culicines in houses with fully closed eaves were similar to those in houses with a single small opening in the eave (RR = 0.86, 95% CI: 0.40–1.88, *Z* = -0.371, *P* = 0.711), fully open eaves (RR = 1.14, 95% CI: 0.52–2.52, *Z* = 0.333, *P* = 0.739), four small openings (RR = 1.17, 95% CI: 0.52–2.62, *Z* = 0.377, *P* = 0.706) and two long sides open (RR = 1.28, 95% CI:0.60–2.75, *Z* = 0.637, *P* = 0.524) (Fig. 5). Pairwise comparisons did not provide evidence that catch sizes of female culicines caught in houses with fully closed eaves were different from those caught in houses with a single small opening in the eave (*Z* = -0.371, Adjusted *P* = 0.987), fully open eaves (*Z* = 0.333, Adjusted *P* = 0.991), four small openings (*Z* = 0.377, Adjusted *P* = 0.986) and two long sides open (*Z* = 0.637, Adjusted *P* = 0.915)).

**Fig. 6:**
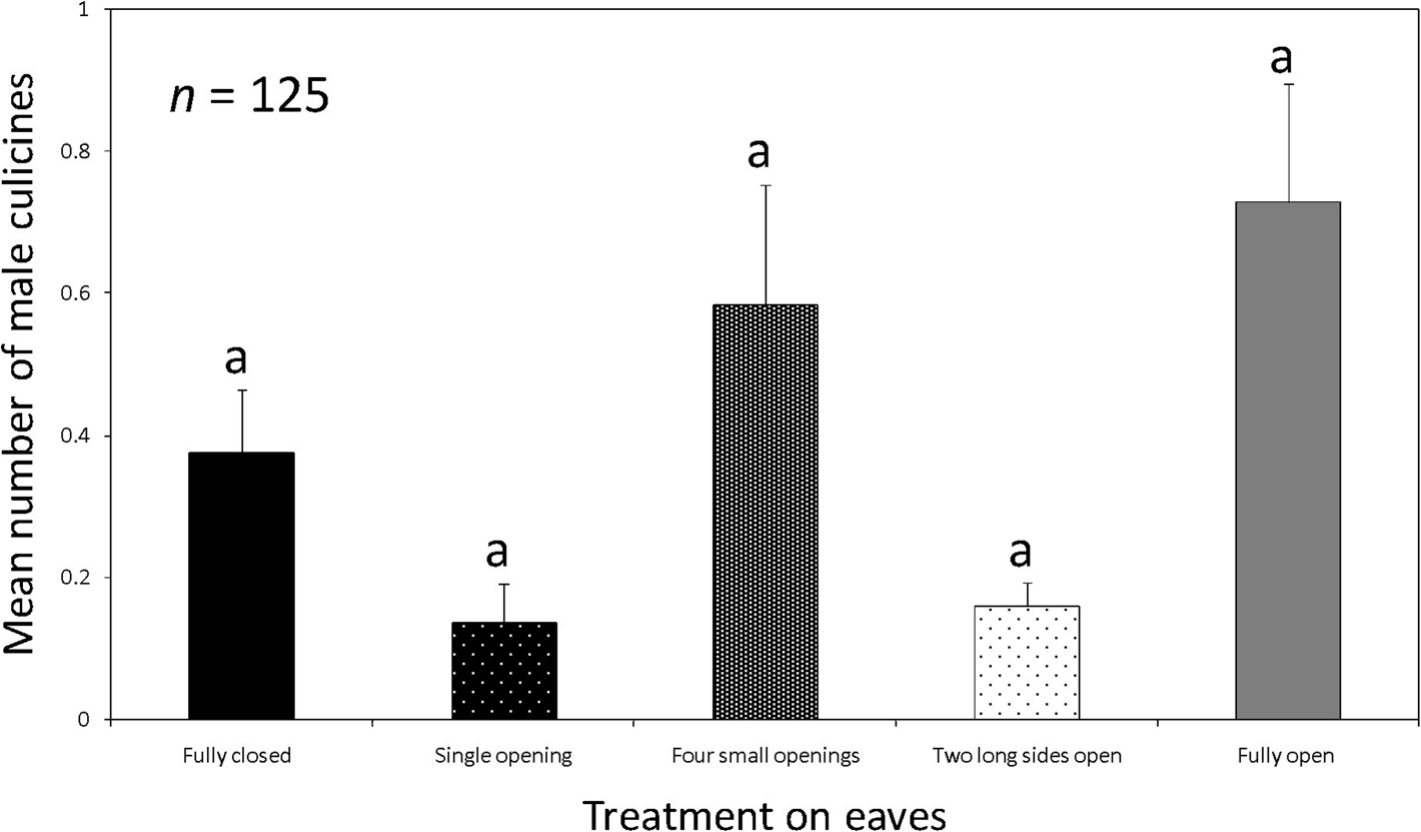
Mean number of male culicines caught indoors with CDC light traps in houses where eaves: were fully closed, had a single small opening, four small openings and fully open. Bars with same letters denote similarities in the number of mosquitoes trapped. *n* = 125 trap nights for each treatment

The proportions of male culicine mosquitoes caught per treatment were: fully closed eaves, 18.95% (*n* = 47); eave with a single small opening, 6.85% (*n* = 17); eave with four small openings, 29.44% (*n* = 73); eave with two long sides open, 8.06% (*n* = 20); and open eaves, 36.69% (*n* = 91). There was no evidence to indicate a significant difference between catch sizes of male culicine mosquitoes in houses with fully closed eaves and houses with other treatments. Pairwise comparison showed that catch sizes of male culicine mosquitoes in houses with fully closed eaves were similar to those in houses with an eave that had one small opening (*Z* = -1.135, Adjusted *P* = 0.609), two long sides open (*Z* = -0.884, Adjusted *P* = 0.785), four small openings (*Z* = -0.370, Adjusted *P* = 0.988) and fully open eaves (*Z* = 0.040, Adjusted *P* = 1.0) (Fig. 6).

Wall type did not have an effect on the number of female anophelines (RR = 0.57, 95% CI: 0.11–2.86, *Z* = -0.681, *P* = 0.496) but the presence of chickens and the number of people who slept in the house the previous night were significantly and positively associated with catches of female anophelines (RR = 4.15, 95% CI: -2.04–8.42, *Z* = 3.938, *P* = 0.001 and RR =1.27, 95% CI: 1.03–1.56, *Z* = 2.263, *P* = 0.024, respectively) (Table 1). The number of people that slept in the house the previous night ranged from one to eight (mean ± SE, 3.41± 0.063). The presence of goats near a house was negatively associated with female culicine catches (RR = 0.70, 95% CI: 0.52–0.94, *Z* = -2.385, *P* = 0.017). Catches of female culicines in houses where people cooked outside, 2m away from the house, were different from those in houses where people cooked on the veranda (RR = 0.63, 95% CI: 0.46–0.87, *Z* = -2.816, *P* = 0.005), but similar to those where people cooked within 2 m of the house (RR = 0.80, 95% CI: 0.62–1.02, *Z* = -1.819, *P* = 0.069) and inside the house (RR = 1.46, 95% CI: 0.98–2.17, *Z* = 1.841, *P* = 0.066). The presence of chickens was negatively associated with the male culicine catches (RR = 0.56, 95% CI: 0.32–0.99, *Z* = -2.002, *P* = 0.045) (Table 1).

**Table 1:** Effect of treatment, livestock, cooking locations, wall type and the number of people that slept in the house the previous night on the catch sizes of anophelines and culicines. The risk ratios (RR) and 95% confidence intervals (CI) are shown

Treatment	Female anophelines		Female culicines		Male culicines	
	RR	95% CI	RR	95% CI	RR	95% CI
Open eaves	14.16	2.05–97.91	1.14	0.52–2.52	1.03	0.21–5.12
Eaves with two long sides open	5.41	0.72–40.40	1.28	0.60–2.75	0.49	0.10–2.37
Eaves with four small openings	8.83	1.16–67.14	1.17	0.52–2.62	0.73	0.14–3.82
Eaves with a single small opening	4.38	0.59–32.46	0.86	0.40–1.88	0.40	0.08–1.94
Fully closed eaves	Ref	–	Ref	–	Ref	–
People that slept in the house the previous night	1.27	1.03–1.56	1.07	0.99–1.15	0.98	0.90–1.07
Cow	0.46	0.10–2.15	1.29	0.96–1.73	0.68	0.44–1.05
Goat	1.16	0.49–2.77	0.70	0.52–0.94	1.04	0.70–1.55
Chicken	4.15	2.04–8.42	0.95	0.69–1.32	0.56	0.32–0.99
Cooking inside the house	2.20	0.66–7.36	1.46	0.98–2.17	0.98	0.52–1.85
Cooking on the veranda	2.34	0.78–7.05	0.63	0.46–0.87	0.85	0.52–1.39
Cooking outside, within 2m of the house	1.04	0.43–2.50	0.80	0.62–1.02	1.13	0.80–1.60
Cooking outside, away from 2m of the house	Ref	–	Ref	–	Ref	–
Wall type fire baked bricks	0.57	0.11–2.86	1.83	0.89–3.75	1.73	0.42–7.13
Wall type sun-dried bricks	Ref	–	Ref	–	Ref	–

*Abbreviation*: *Ref* reference

## Discussion

Houses with fully closed eaves had reduced rates of house entry by anopheline mosquitoes compared to houses with fully open eaves, similar to findings from other regions in Africa [8, 16, 37]. The reduced number of anophelines indoors suggests that a house improvement package that includes fully closed eaves could serve as an effective malaria intervention by reducing vector-human contact. Houses with fully closed eaves also had fewer malaria mosquitoes than houses with four small openings in the eaves, indicating that the latter group of houses would not provide the same level of protection against bites from malaria vectors as would houses with fully closed eaves. Malaria vectors were likely able to locate the small gaps in the eaves (i.e. the experimental sub-optimal modifications) due to the concentration of airflow and host odours emanating through such small gaps [18]. In fact, the ability of mosquitoes to readily find these holes is being exploited by studies looking at the impact of eave tubes on mosquito populations, whereby small sections of PVC tubing fitted with electrostatic netting that is treated with powdered insecticide or entomopathogenic fungi are inserted along closed eaves [38, 39]. Small, uncovered openings in the eaves, such as those used as experimental treatments in the current study, may reduce the effectiveness of house improvement as a malaria intervention because malaria mosquitoes would still find their way into the house [40].

While fully-closed eaves clearly reduced the number of mosquitoes in the house, we still collected a few malaria vectors, and a considerable number of culicines, in those houses. The most probable explanation is that the mosquitoes entered through the doors [15]. While the doors on all of the houses were modified so that mosquitoes could not enter when the doors were closed, we could not control when the doors were closed. Many residents shut their doors late in the evening, facilitating the entry of mosquitoes, especially for crepuscular species. Further research is needed into the behaviour of mosquitoes around doors, and the effect of door modifications for vector-borne disease control.

The study was carried out in traditional houses spread across four villages (about 2 km) allowing for comparisons among different levels of eave closure under natural conditions. While inclusion criteria were used to increase comparability among the houses, we included in our analysis additional factors that may have influenced the entry of mosquitoes into houses. Similar to previous studies, the number of people who slept in the house the previous night was associated with significantly higher numbers of female malaria vectors indoors [41–43]. In the current study, the presence of chickens within 20m of the house was also associated with more female anophelines indoors, most of which were *Anopheles arabiensis*. This concurs with the findings of a semi-field study using chicken odour in Kenya [44], but differs with the findings of Jaleta et al. [45], who found that chickens or chicken volatiles reduced the catches of female *An. arabiensis* mosquitoes .The relationship between chicken odours and anopheline mosquitoes warrants further investigation.

Presence of goats and cooking on the veranda was associated with reduced female culicines. Interestingly, cooking on the veranda was also associated with reduced male culicine catches. Male mosquitoes feed on sugar and do not seek hosts for blood, but this factor was also associated with the female culicines. It is possible that the males could have been using these odour cues to locate likely presence of female culicines, an area that needs further investigation.

The relatively low number of mosquitoes collected during this study can probably be attributed to two factors. First, the rainy season prior to the study (November 2015 to April 2016) was relatively dry, with drought conditions throughout the region, and Chikhwawa District specifically receiving extremely below average rainfall [46]. Additionally, the National Malaria Control Programme in Malawi conducted a mass distribution of ITNs in April 2016. Both factors likely reduced the mosquito populations in the study area.

Observational studies assessing the impact of housing on malaria have consistently found that people living in houses with modern features, such as closed eaves, have lower odds of malaria infection [24], even when accounting for ITN use [47].These findings have increased international interest in house improvement as a deliberate intervention against malaria [24]. House improvement covers and protects all individuals sleeping in a house equally, and its impact should not be affected by insecticide resistance. Still, observational studies are considered low-quality evidence with a high risk of bias. An ongoing trial in the Gambia aims to assess the impact of house improvement on the incidence of clinical malaria using a randomised design [48]. An ongoing cluster randomised trial in Malawi is evaluating the impact of house improvement, using a community-led implementation approach, on malaria transmission [26]. The results of the current study indicate that the quality of eave closure will be one of the important coverage indicators for understanding the effects of house improvement in these ongoing trials.

## Conclusions

Our study adds to the evidence that house improvement, including fully closed eaves, reduces the number of malaria vectors indoors and, therefore, shows promise as a complementary tool for malaria control. While further research is necessary to understand the behaviour of malaria vectors around house entry points, the results of this study demonstrate the ability of malaria vectors to locate any remaining entry points on improved houses, suggesting that quality control must be an important component of implementing house improvement as an intervention [2]. The lack of effect on culicine mosquitoes in this study could reduce acceptance of house improvement, as implemented here, by household residents due to continued nuisance biting. This limitation could be addressed through community engagement (e.g. encouraging people to close their doors early in the evenings) or improved designs.

## Abbreviations

ITNs: Insecticide-treated bed nets
IRS: Indoor residual spraying
CDC: Centers for Disease Control and Prevention
